# Omentectomy for oncological surgical staging by transvaginal natural orifice transluminal endoscopic surgery (vNOTES): a preliminary study

**DOI:** 10.3389/fsurg.2023.1224770

**Published:** 2023-07-27

**Authors:** Yannick Hurni, Daniela Huber

**Affiliations:** ^1^ Department of Gynecology and Obstetrics, Valais Hospital, Sion, Switzerland; ^2^ Department of Pediatrics, Gynecology and Obstetrics, Geneva University Hospitals, Geneva, Switzerland

**Keywords:** omentectomy, ovarian cancer, endometrial cancer, natural orifice transluminal endoscopic surgery (NOTES), surgical staging

## Abstract

**Objective:**

This study aimed to determine the feasibility of performing omentectomy by transvaginal natural orifice transluminal endoscopic surgery (vNOTES) for surgical staging of ovarian and high-risk endometrial malignancies.

**Methods:**

This descriptive study was realized in a non-university hospital in Switzerland. Eighteen patients with suspicious adnexal masses or high-risk endometrial cancer underwent surgical staging comprising infracolic omentectomy by vNOTES between May 2020 and April 2023.

**Results:**

Patients underwent oncological surgical staging for suspicious adnexal masses in 14 cases (77.8%) and high-risk endometrial cancer in 4 cases (22.2%). vNOTES omentectomies were performed in all patients without complications. Associated procedures included salpingo-oophorectomy (94.4%), hysterectomy (55.6%), peritoneal biopsies (33.3%), pelvic sentinel lymph node biopsies (22.2%), and appendectomy (5.6%). The median time to perform omentectomies was 9 (4–13) min. All oncological staging were completed by vNOTES. No significant intraoperative complications occurred. We observed 1 case (5.6%) of postoperative fever probably associated with vaginal cuff infection (Clavien-Dindo grade II).

**Conclusions:**

This study demonstrated the feasibility of performing vNOTES oncological staging requiring complex extrapelvic procedures such as infracolic omentectomy, supporting its potential role for managing gynecological malignancies such as ovarian and high-risk endometrial cancers. However, before expanding this approach outside study settings, strong evidence of its feasibility, practical benefits, and long-term oncological outcomes are needed.

## 1. Introduction

Infracolic omentectomy is an essential part of the surgical staging for ovarian and high-risk endometrial cancers (1, 2). This procedure can be performed by laparotomy, conventional laparoscopy, laparo-endoscopic single-site, robotic multiport, and robotic laparo-endoscopic single-site (3–6). Increasing evidence supports the safety and efficiency of minimally invasive procedures in managing early-stage ovarian and endometrial malignancies (7–11).

In recent years, transvaginal natural orifice transluminal endoscopic surgery (vNOTES) has been studied as a new minimally invasive approach to treating gynecological malignancies (12, 13). Compared to other surgical approaches, vNOTES seems to present reduced blood loss, less postoperative pain, and shorter hospitalization length (14, 15). Although increasing evidence supports the use of this technique to manage early-stage endometrial cancer (16–18) and to perform benign adnexal surgeries (14, 19, 20), little is known about the feasibility of performing vNOTES oncological staging requiring complex extrapelvic procedures such as infracolic omentectomy.

In the present study, we report our experience performing vNOTES omentectomy for surgical staging for suspicion of ovarian or high-risk endometrial malignancies.

## 2. Materials and methods

### 2.1. Patient selection, data collection, and methods

Since May 2020, vNOTES has been introduced in our institution (Valais Hospital in Sion, Switzerland). From January 2022, we started collecting, both retrospectively and prospectively, data concerning patients who underwent vNOTES procedures to create an institutional database. This database was created using Research Electronic Data Capture (REDCap) software. All patients gave written informed consent, and the project received approval from the local ethical committee (CER-VD), with registration number 2021-02346. From this database, we identified all patients who had undergone a vNOTES omentectomy as part of a surgical staging for suspicion of ovarian or high-risk endometrial malignancies.

Patients were selected for vNOTES procedures based on preoperative workups suggesting an early disease stage. Patients with suspicious adnexal masses underwent preoperative pelvic ultrasound sometimes associated with pelvic magnetic resonance imaging, thoracic-abdominopelvic computed tomography scan, and CA-125 tumor marker assessment. Patients with high-risk endometrial malignancies underwent preoperative pelvic ultrasound, pelvic magnetic resonance imaging, and thoracic-abdominopelvic computed tomography scan. We excluded patients with suspicion of advanced-stage disease, a history of perineal/rectal surgery, a history of pelvic radiation, suspected rectovaginal or retrocervical endometriosis, or active pelvic inflammatory disease.

Demographic features, as well as clinical and perioperative information, were collected and analyzed. Intraoperative data included total operating time (time from the initial incision to vaginal closure), time to insert the vNOTES port, omentectomy time, blood loss, intraoperative complications, and the need to convert to conventional laparoscopy or laparotomy. Postoperative data included pain evaluation with the visual analog scale (VAS) graded from 0 to 10 at 12, 24, and 48 h after surgery, the use of opioid analgesics, complications that occurred up to 3 months after surgery, and final histopathological diagnosis with the International Federation of Gynecology and Obstetrics (FIGO) stage for confirmed malignancies. Postoperative complications were graded using the Clavien-Dindo classification (CD) (21).

The primary outcome was the feasibility of performing a vNOTES omentectomy. Secondary outcomes included (1) the feasibility of performing a complete oncological surgical staging as planned; (2) the duration of the entire surgery, the time to install the vNOTES platform, and the time to perform the omentectomy; (3) the intraoperative complications rate and type; (4) the conversion rate to conventional laparoscopy or laparotomy; (5) the postoperative complications rate, type, and assessment using the CD classification; (6) the length of hospital stay in days; and (7) the postoperative pain evaluation using a VAS and by the postoperative use of opioid analgesics.

Continuous variables were presented as median and range. Dichotomous variables were presented as absolute numbers and percentages (%). Statistical analysis was performed using IBM SPSS version 20 (IBM, Corp., Armonk, NY, USA).

### 2.2. Surgical technique

Patients were placed in a dorsal lithotomy position under general anesthesia and received prophylactic intravenous antibiotics with cefuroxime 1.5 g and metronidazole 500 mg. A Foley catheter was placed to keep the bladder empty. Access to the peritoneal cavity was gained through anterior and posterior colpotomy when concomitant hysterectomy was performed, and posterior colpotomy only in the case of uterus-preserving procedures. A vNOTES port (GelPoint vPath, Applied Medical, Rancho Santa Margarita, CA, USA) was placed in the abdominal cavity. Carbon dioxide was insufflated to create a pneumoperitoneum with an intraperitoneal pressure of 10–15 mmHg. Three trocars were used to insert a 10 mm rigid 30° camera, 5 mm instruments such as Johan and bipolar graspers, and an articulating sealing device. We carefully inspected the uterus, adnexa, and all peritoneal surfaces to check for eventual tumor spread. We performed peritoneal washing collection, uni- or bilateral salpingo-oophorectomy, total hysterectomy, peritoneal biopsies, appendectomy, and pelvic sentinel lymph node biopsies by each case's specifics. Pelvic sentinel lymph node biopsies were performed for patients presenting with endometrial cancer following our previously described technique (16). Specimens were extracted through the vagina. An Alexis Contained Extraction System (Applied Medical, Rancho Santa Margarita, CA, USA) was used to remove the adnexa and for large uteri requiring a vaginal morcellation. Intraoperative frozen section analysis was performed in all cases of suspicious adnexal masses. Infracolic omentectomy was performed for results suggesting adnexal malignancy or inconclusive intraoperative pathologic results in patients expressively requesting to minimize the risk of reinterventions for incomplete staging. To perform the infracolic omentectomy (see Supplementary Video S1), the patient was kept in the Trendelenburg position. The greater omentum was grasped and lifted to expose its attachment to the transverse colon. Dissection was realized with an articulating sealing and cutting device, separating the greater omentum from the transverse colon. Dissection was realized starting from the right through the left or inversely depending on the case's specifics. The omentum was extracted through the vagina. The vaginal cuff was closed under direct vision with a running suture using Vicryl 0. Clindamycin vaginal cream was administered once a day on the evening before the surgery, the day of the surgery, and for the first 7 postoperative days.

## 3. Results

Between May 2020 and April 2023, 18 patients underwent vNOTES omentectomy for oncological staging. All patients were operated on by the same surgeon (DH). Tables 1, 2 summarize the patient's demographic characteristics and indications for surgery. The median age was 59 (30–81) years, and the median body mass index was 23.8 (16.0–39.2) kg/m^2^. Patients underwent oncological surgical staging for suspicious adnexal masses at ultrasound exams in 14 cases (77.8%) and for high-risk endometrial cancer at endometrial biopsies in 4 cases (22.2%).

**Table 1:** Demographic data and medical history.

Age (y)	59 (30–81)
BMI (Kg/m^2^)	23.8 (16.0–39.2)
BMI >30 Kg/m^2^	4 (22.2)
Previous vaginal delivery	12 (66.7)
Previous cesarean section	4 (22.2)
Previous pelvic/abdominal surgery	12 (66.7)

Data are presented as median and range or number and percentage.

BMI, body mass index.

**Table 2:** Indications for surgery, surgical procedure and postoperative diagnosis.

Patient	Indication for surgery	Surgical procedure	Postoperative histopathological diagnosis
1	Suspicious right adnexal mass (4 cm)	VANH, bilateral salpingo-oophorectomy, infracolic omentectomy, peritoneal biopsies, peritoneal washing	Low-grade serous ovarian cancer (FIGO IIB)
2	Suspicious right adnexal mass (4 cm)	VANH, bilateral salpingo-oophorectomy, infracolic omentectomy, peritoneal washing	Ovarian tecoma
3	Suspicious right adnexal mass (15 cm)	Right salpingo-oophorectomy, infracolic omentectomy, peritoneal washing	Ovarian cystoadenofibroma
4	Suspicious right adnexal mass (5 cm)	VANH, bilateral salpingo-oophorectomy, infracolic omentectomy, peritoneal biopsies, peritoneal washing	Ovarian adult granulosa cell tumor (FIGO IIB)
5	Suspicious right adnexal mass (14 cm)	Right salpingo-oophorectomy, infracolic omentectomy, appendectomy, peritoneal washing	Ovarian seromucinous cystadenoma
6	Suspicion of right ovarian borderline tumor (7.5 cm)	VANH, bilateral salpingo-oophorectomy, infracolic omentectomy, appendectomy, peritoneal washing	Mucinous borderline ovarian tumor (FIGO IA)
7	Suspicion of right ovarian borderline tumor (3.5 cm)	Right salpingo-oophorectomy, infracolic omentectomy, peritoneal washing	Low-grade endometrioid ovarian tumor (FIGO IA)
8	Suspicious left adnexal mass (7 cm)	VANH, bilateral salpingo-oophorectomy, infracolic omentectomy, peritoneal biopsies, peritoneal washing	Bilateral benign ovarian Brenner tumors
9	Suspicious left adnexal mass (4.5 cm)	Bilateral salpingo-oophorectomy, Infracolic omentectomy, peritoneal biopsies, peritoneal whashing	Ovarian adult granulosa cell tumor (FIGO IA)
10	Suspicious left adnexal mass (5 cm)	Bilateral salpingo-oophorectomy, infracolic omentectomy, peritoneal washing	Ovarian serous cystadenoma
11	Endometrial carcinoma with sarcomatous components on endometrial biopsy	VANH, bilateral salpingo-oophorectomy, infracolic omentectomy, peritoneal washing	Endometrial carcinosarcoma (FIGO IIIA)
12	Endometrial clear cell carcinoma on endometrial biopsy	VANH, bilateral salpingo-oophorectomy, infracolic omentectomy, peritoneal washing, bilateral pelvic sentinel lymph node biopsy	Endometrial clear cell carcinoma (FIGO IB)
13	Endometrial clear cell carcinoma on endometrial biopsy	VANH, bilateral salpingo-oophorectomy, infracolic omentectomy, peritoneal washing, bilateral pelvic sentinel lymph node biopsy	Endometrial clear cell carcinoma (FIGO IA)
14	Suspicion of left ovarian borderline tumor (4 cm)	VANH, bilateral salpingo-oophorectomy, infracolic omentectomy, peritoneal washing	Ovarian serous cystadenoma
15	Suspicious right adnexal mass (3 cm)	Right salpingo-oophorectomy, infracolic omentectomy, peritoneal washing	Ovarian cystadenofibroma
16	Endometrial mixed cell adenocarcinoma	VANH, bilateral salpingo-oophorectomy, infracolic omentectomy, peritoneal washing, bilateral pelvic sentinel lymph node biopsy	Endometrial mixed cell adenocarcinoma (FIGO IA)
17	Suspicious right adnexal mass (5 cm)	Right salpingo-oophorectomy, infracolic omentectomy, peritoneal biopsies, peritoneal washing	Ovarian Dermoid Tumor
18	Suspicious left adnexal mass (9 cm)	Left salpingo-oophorectomy, infracolic omentectomy, peritoneal biopsies, peritoneal washing	Mucinous borderline ovarian tumor (FIGO IA)

VANH, vaginal assisted NOTES hysterectomy; FIGO, international federation of gynecology and obstetrics.

Intraoperative characteristics are summarized in Table 3. vNOTES omentectomies were performed in all patients without any complications. Associated procedures included salpingo-oophorectomy (18/18, 100.0%), hysterectomy (10/18, 55.6%), peritoneal biopsies (6/18, 33.3%), pelvic sentinel lymph node biopsies (4/18, 22.2%), and appendectomy (2/18, 11.1%). The median operative time was 80 (35–178) min, with a median time to install the vNOTES port of 12 (7–25) min and a median time to perform omentectomies of 9 (4–13) min. All oncological staging were completed as planned by vNOTES. No significant intraoperative complications occurred, and the median estimated blood loss was 50 (10–150) ml. In one case (5.6%), a 1 cm rectal serosal tear was observed after vNOTES port insertion. The lesion was repaired with an absorbable suture, and the patient presented no subsequent problems. Conversion to conventional laparoscopy or laparotomy was never necessary. In one case, a hybrid approach combining vNOTES with a transumbilical trocar was used to perform a salpingo-oophorectomy for a 17 cm ovarian lesion, allowing secure transabdominal specimen extraction and reducing the risk of tumor cell spilling. We decided to opt for this hybrid approach intraoperatively, and the transumbilical trocar was positioned after conventional vNOTES abdominal access.

**Table 3:** Surgical procedure and operative characteristics.

Procedures performed	
Infracolic omentectomy	18 (100)
Salpingo-oophorectomy	18 (100)
Unilateral	5 (27.8)
Bilateral	12 (66.7)
Largest diameter (mm)	48 (35–150)
vNOTES hysterectomy	10 (55.6)
Uterine weight (g)	130 (47–905)
Peritoneal biopsies	6 (33.3)
Bilateral pelvic sentinel lymph node biopsy	4 (22.2)
Appendectomy	2 (11.1)
Extensive adhesiolysis	7 (38.9)
Operative time	80 (35–178)
vNOTES port insertion (min)	12 (7–25)
Omentectomy (min)	9 (4–13)
Estimated blood loss (ml)	50 (10–150)
Specimens retrieval	
Transvaginal	17 (94.4)
Morcellation into a bag	7 (38.9)
Transabdominal	1 (5.6)
Conversion to conventional laparoscopy/laparotomy	–
Intraoperative complications	1 (5.6)
* *Rectal serosal tear	1 (5.6)

Data are presented as median and range or number and percentage.

vNOTES, transvaginal natural orifice transluminal endoscopic surgery.

Interventions were performed as one-day surgery in 3 cases (16.7%), while 15 patients (83.3%) remained hospitalized for a median time of 2 (1–5) days. Median postoperative VAS was 0.5 (0–5), 3 (0–4), and 1(0–4) at 12 h, 24 h, and 48 h after surgery, respectively. Postoperative opioids were never necessary. We observed no postoperative complications during hospitalizations. In one case (6.7%), pelvic infection without vaginal cuff dehiscence was suspected in a patient presenting with postoperative fever one week after the surgery. No re-intervention was necessary, and the patient fully recovered with enteral antibiotics (CD classification grade II). Final histopathological diagnoses and postoperative outcomes are summarized in Tables 2, 4, respectively.

**Table 4:** Postoperative outcomes.

Surgery regimen	
One-day surgery	3 (16.7)
Hospitalization	15 (83.3)
Length of stay (days)	2 (1–5)
Pain visual analog scale (1–10)	
12 h postoperative	0.5 (0–5)
24 h postoperative	3 (0–4)
48 h postoperative	1 (0–4)
Use of opioids during the postoperative period	–
Postoperative complications	1 (5.6)
Wound infection	1 (5.6)

Data are presented as median and range or number and percentage.

## 4. Discussion

In recent years, vNOTES have been gaining popularity in benign gynecological surgery, and increasing data suggest its role as a valuable alternative to conventional laparoscopy (14, 15). However, its potential role in managing gynecological malignancies is still under investigation. In patients with early-stage endometrial cancer, vNOTES surgical staging with sentinel lymph node biopsy appeared feasible and potentially advantageous compared to the conventional laparoscopic approach (16, 17). Although vNOTES adnexal surgery appears feasible (19, 20), limited data exist concerning oncological staging for ovarian malignancies (12, 13). This reluctance could be associated with potential risks of tumor cell spilling in managing adnexal masses and concerns about the difficulty of performing complex extrapelvic procedures such as infracolic omentectomy.

In 2020, Lowenstein et al. first reported the first 5 cases of successful vNOTES staging, including omentectomy for ovarian malignancies (12). Since that moment, no more cases have been reported other than those described by our group. In the present study, we report our initial experience with vNOTES omentectomy for the surgical staging of ovarian and high-risk endometrial malignancies. In 18 consequent cases, we could perform the infracolic omentectomy and all other planned surgical steps by vNOTES. Conversion to conventional laparoscopy or laparotomy was never required, and we observed no significant perioperative complications. The median time of 9 (5–13) min was comparable to previously reported laparoscopic and vNOTES omentectomies (12, 22). These results demonstrate the feasibility and safety of vNOTES omentectomy, suggesting that this surgical approach could be an alternative to conventional laparoscopy in the surgical staging of ovarian and high-risk endometrial cancers. However, careful patients selection, including medical history, clinical examination, and complementary preoperative radiological exams such as thoracic-abdominopelvic ultrasound, magnetic resonance imaging, and/or computed tomography scan, should be performed to minimize the risk of perioperative complications, intraoperative tumor cell spilling, and of missing eventual advanced-stage diseases.

The main advantages of vNOTES compared to conventional laparoscopy are associated with its natural access route through the vagina, leaving no visible scars and reducing postoperative pain. Other potential benefits are reduced blood loss, no complications of abdominal accesses, and shorter hospitalization time. All these improve postoperative recuperation and patient satisfaction, making vNOTES interesting for the management of oncological patients, for whom one should pay particular attention to postoperative recovery and timing to eventual adjuvant chemo-/radiotherapy.

Limitations of vNOTES include reduced instrument triangulation, restricted space for manipulations, and limited accessibility of some anatomical regions. In oncological surgical staging, this affects the ability to inspect some peritoneal areas satisfactorily, such as the prevesical peritoneum, costodiaphragmatic recesses, Morrison's pouch, and intestinal mesentery, and to perform extrapelvic procedures such as peritoneal biopsies or infracolic omentectomy. Using articulating instruments and variable-view rigid endoscopes can help overcome these constraints (23). Transvaginal access and restricted spaces concern inadvertent tumor cell spilling, especially when manipulating adnexal masses and extracting large uteri. Delicate manipulations and specimen extractions should be performed into specific endobags to reduce the risk of contamination (24, 25). In addition, hybrid approaches combining vNOTES and transabdominal ports, which allow different visual and instrumental access methods, could improve safety in managing very large adnexal lesions hardly operable by conventional laparoscopy or vNOTES alone.

This study supports vNOTES as a feasible and safe technique for performing surgical staging in ovarian and endometrial malignancies, even when infracolic omentectomy is indicated. We acknowledge some limitations of this study, mainly resulting from its single-institution character and the limited number of patients included in the analyses. However, these preliminary results are essential for developing the vNOTES in gynecological malignancies. Although surgical feasibility and safety seem increasingly evident, more studies are needed to prove vNOTES long-term oncological safety.

### 4.1. Conclusions

This study demonstrated the feasibility of performing vNOTES oncological staging requiring complex extrapelvic procedures such as infracolic omentectomy, supporting its potential role for managing gynecological malignancies such as ovarian and high-risk endometrial cancers. However, before expanding this approach outside study settings, strong evidence of its feasibility, practical benefits, and long-term oncological outcomes are needed.

## Data Availability

The raw data supporting the conclusions of this article will be made available by the authors, without undue reservation.

## Supplementary material

Supplementary Video S1 Infracolic omentectomy by vNOTES. In this video, we demonstrate the use of a vNOTES approach to perform omentectomy.
